# Dietary carbohydrates influence muscle texture of olive flounder *Paralichthys olivaceus* through impacting mitochondria function and metabolism of glycogen and protein

**DOI:** 10.1038/s41598-020-76255-3

**Published:** 2020-12-11

**Authors:** Jiahuan Liu, Kangyu Deng, Mingzhu Pan, Guangxia Liu, Jing Wu, Mengxi Yang, Dong Huang, Wenbing Zhang, Kangsen Mai

**Affiliations:** 1 The Key Laboratory of Aquaculture Nutrition and Feeds (Ministry of Agriculture and Rural Affairs), the Key Laboratory of Mariculture (Ministry of Education), Ocean University of China, Qingdao, 266003 China; 2 Laboratory for Marine Fisheries Science and Food Production Processes, Qingdao National Laboratory for Marine Science and Technology, Wen Hai Road, Qingdao, 266237 China; 3 Shenzhen Alpha Group Co., Ltd., Shenzhen, China

**Keywords:** Biochemistry, Molecular biology, Zoology

## Abstract

The present study was conducted to estimate the effects of dietary carbohydrates on muscle quality and the underlying mechanisms. Six isonitrogenous and isolipidic diets were formulated to contain graded levels of carbohydrates (0%, 8%, 12%, 16%, 20% and 24%, respectively). These diets were named as C0, C8, C12, C16, C20 and C24, respectively. After a 10-week feeding trial, results showed that the muscle pH, liquid holding capacity (LHC) and hardness were significantly decreased by the increasing dietary carbohydrate levels. Dietary carbohydrates significantly decreased the muscle fibre diameter, and the highest value was found in the C0 group. Accumulated glycogen and degenerated mitochondrial cristae were observed in the C24 group. Significantly higher contents of protein carbonyls were observed in the C20 group and C24 group (*P* < 0.05). There was a significant decrease of mtDNA copy number in the C24 group compared with that in the C0 and C8 groups. The AMP/ATP ratio in muscle decreased first and then increased with the increasing dietary carbohydrate levels. The dietary incorporation of carbohydrate significantly reduced the expression of *opa1*, *pygm* and genes involved in myogenesis (*myf5* and *myog*). Meanwhile, proteolysis-related genes (*murf-1*, *mafbx*, *capn2* and *ctsl*), pro-inflammatory cytokines (*il-6* and *tnf-α*) and *mstn* were significantly up-regulated. In the C24 group, significant increase of phosphorylation of AMPK (Thr172), up-regulation of PGC-1α and GLUT4 were observed, while the phosphorylation level of S6 (Ser235/236) was significantly decreased. It was concluded that excessive dietary carbohydrate level (24%) had negative impacts on mitochondria function and promoted glycogen accumulation, and thereafter influenced the muscle quality of olive flounder. The activation of AMPK as well as the upregulation of PGC-1α and GLUT4 was the key mechanism.

## Introduction

Carbohydrates are often the most economical source of dietary energy and can be supplied in large quantities. The sustainability of aquaculture and the approach to conduct aquaculture in an environmentally responsible way are the concerns for both the consumer and the fish farmer^1^. Appropriate dietary carbohydrate level resulted in a better growth performance and reduced the catabolism of proteins and lipids in fish^2–4^. The incorporation of carbohydrate in fish diets can help to ease the tightening supply of fish meal and fish oil. Moreover, the introduction of carbohydrate can reduce the ammonia excretion by amino acid metabolism^5^. It is helpful to the long-term sustainability of aquaculture. However, fish, especially carnivorous fish, is generally considered to have a poor utilization ability of dietary carbohydrates^6,7^. In addition to the reduced growth performance and poor physiological functions^3,8,9^, change of muscle quality can also be led by excessive dietary carbohydrate in fish^10^. The understanding of the mechanism behind these phenomenons can help the better application of carbohydrate in aqua-feed.

Flesh quality is a complex concept and usually defined in terms of texture, taste, smell, juiciness and appearance^11^. Fish quality is receiving increasing attention as aquaculture production and demand increase. Muscle texture is important for consumers’ acceptability of fish. The muscle fibre cellularity, connective tissue, proteolysis and nutrient storage are decisive factors of the flesh texture^12–15^. Previous studies have identified some genes associated with the muscle cellularity of fish, such as insulin-like growth factors (IGFs), myostatin (*mstn*) and myogenic regulatory factors (MRFs)^15–17^. The molecular mechanisms on the fiber size distribution have the potential to change the energy allocation and texture during growth, which will influence the eating quality of the flesh^18^.

Muscle growth was proved to be affected by regulation of protein content in the body^19^. The growth and maintenance of the skeletal muscle depends mainly on the balance between catabolic and anabolic metabolism of the protein, which in turn affects the muscle cellularity^20^. The mammalian target of rapamycin (mTOR) is a crucial factor, which regulates protein synthesis and stimulates cell growth in a nutrient-sensitive signaling^21^. In fish muscle, there are mainly three protein degradation systems including ubiquitin–proteasome system, calpain proteases and lysosomal cathepsins^19,22^. These three protein degradation systems are involved in body protein turnover and promote postmortem muscle softening.

Previous studies showed that muscle quality can be affected by dietary carbohydrates^23–25^. Higher dietary carbohydrate levels (44.1% vs 30.9%) increased the glycogen concentration together with postmortem glycolytic potential in muscle by influencing glycolytic enzymes activities and myofibre transformation^23,26,27^. It led to lower pH, higher drip loss, tender texture and paler color of pig muscle^23,25^. In fish, it was found that higher dietary carbohydrate levels (28% vs 4%) caused higher glycogen content and lower hardness in muscle of dentex (*Dentex dentex*)^27^. Carbohydrate higher than 27% in diet increased total volatile bases nitrogen, thiobarbituric acid and free fatty acids, which had effects on fillet quality of beluga (*Huso huso*) during frozen storage^28^. However, the mechanism of effects of dietary carbohydrate on the muscle growth and quality is still not clarified in fish.

Olive flounder (*Paralichthys olivaceus*), a carnivorous marine fish, is one of the most commercially important aquaculture fish species in China. A previous study has reported that the optimal dietary carbohydrate content for the growth of olive flounder is 15.8%^29^. Generally, the amount of carbohydrates included in diets for carnivorous species is < 20%^30–32^. In the present study, six experimental diets with wide range of carbohydrate levels (0%, 8%, 12%, 16%, 20% and 24%, respectively) were formulated to investigate the effect of graded levels of dietary carbohydrate, including the deficient (0%) and the excessive (24%), on the muscle quality and the underlying mechanisms in olive flounder.

## Results

### Survival and growth performance

There were no significant differences in the survival rate (94.22–95.56%) and feed efficiency ratio (1.38–1.42) of olive flounder among all the treatments after the feeding trial (*P* < 0.05). The specific growth rate in the group of C16 (3.65) was significantly higher than those in the groups of C0 (3.57), C8 (3.42) and C24 (3.52) (*P* < 0.05) (Supplementary Table S1)^33–35^.

### Muscle pH and liquid holding capacity (LHC)

Values of muscle pH and LHC are shown in Table 1. The muscle pH values of fish in the C20 group and C24 group were significantly lower than that in the C0 group (*P* < 0.05). Water loss, lipid loss and liquid loss of fish in the C24 group were significantly higher than those in the other groups (*P* < 0.05).

**Table 1:** The pH and liquid holding capacity (LHC) in the muscle of olive flounder after a 10-week feeding trial.

	Dietary carbohydrate level, % dry matter					
	0	8	12	16	20	24
pH	6.74 ± 0.08^b^	6.51 ± 0.06^ab^	6.59 ± 0.06^ab^	6.56 ± 0.03^ab^	6.40 ± 0.05^a^	6.38 ± 0.03^a^
Liquid loss (%)	10.92 ± 0.31^a^	11.07 ± 0.53^a^	10.55 ± 0.63^a^	11.04 ± 0.39^a^	11.84 ± 0.32^a^	14.17 ± 0.51^b^
Water loss (%)	9.60 ± 0.36^a^	9.75 ± 0.44^a^	9.23 ± 0.52^a^	9.89 ± 0.37^a^	10.45 ± 0.25^a^	12.31 ± 0.49^b^
Lipid loss (%)	1.33 ± 0.07^a^	1.32 ± 0.12^a^	1.33 ± 0.13^a^	1.15 ± 0.03^a^	1.39 ± 0.09^ab^	1.80 ± 0.07^b^

All data were expressed as mean ± SE. Mean values within the same row with different superscripts are significantly different (*P* < 0.05; Tukey's test).

### Muscle texture

Data on muscle texture is shown in Table 2. The hardness was significantly decreased by the increasing dietary carbohydrate levels (*P* < 0.05). The lowest value of hardness was found as 78.09 g in the C24 group. Fish in the C20 and C24 groups had significantly lower springiness than that in the C16 group (*P* < 0.05). The chewiness was also significantly (*P* < 0.05) decreased with the increasing of dietary carbohydrate levels. There were no significant differences in cohesiveness among all the treatments (*P* > 0.05). Fish in the C24 group had a significantly lower adhesiveness of muscle than that in the C0 group (*P* < 0.05).

**Table 2:** The muscle texture of olive flounder after a 10-week feeding trial.

	Dietary carbohydrate levels, % dry matter					
	0	8	12	16	20	24
Hardness (g)	121.25 ± 1.01^c^	118.39 1 ± 9.31^bc^	119.51 ± 5.06^bc^	90.44 ± 9.52^abc^	80.74 ± 5.14^ab^	78.09 ± 6.13^a^
Springiness (mm)	0.78 ± 0.04^ab^	0.79 ± 0.08^ab^	0.92 ± 0.04^b^	0.74 ± 0.06^ab^	0.68 ± 0.05^a^	0.69 ± 0.03^a^
Chewiness (mJ)	58.44 ± 1.82^c^	53.20 ± 5.28^bc^	57.10 ± 1.85^bc^	41.58 ± 4.52^b^	25.48 ± 0.57^a^	25.96 ± 3.08^a^
Cohesiveness	0.41 ± 0.00	0.42 ± 0.08	0.43 ± 0.03	0.41 ± 0.07	0.41 ± 0.12	0.41 ± 0.01
Adhesiveness (g × mm)	5.80 ± 0.37^b^	5.41 ± 0.36^ab^	5.57 ± 0.38^ab^	5.32 ± 0.55^ab^	4.77 ± 0.26^ab^	4.01 ± 0.16^a^

All data were expressed as mean ± SE. Mean values within the same row with different superscripts are significantly different (*P* < 0.05; Tukey's test).

### Muscle composition and fibre diameters

Contents of moisture and crude lipid in muscle were not significantly influenced by dietary carbohydrate levels (*P* > 0.05) (Table 3). The crude protein in muscle of fish in the C24 group was significantly decreased compared with those in the other groups (*P* < 0.05). On the contrary, the glycogen content in muscle in the C24 group was significantly higher than those in the C0, C8 and C12 groups (*P* < 0.05).

**Table 3:** Muscle composition and cellularity of olive flounder after a 10-week feeding trial.

	Dietary carbohydrate levels, % dry matter					
	0	8	12	16	20	24
**Muscle composition (wet weight)**						
Moisture (%)	76.43 ± 0.46	76.64 ± 0.27	76.63 ± 0.22	76.57 ± 0.30	76.56 ± 0.23	76.71 ± 0.26
Crude lipid (%)	1.00 ± 0.08	0.90 ± 0.05	0.97 ± 0.13	1.06 ± 0.08	1.23 ± 0.07	1.27 ± 0.13
Crude protein (%)	21.39 ± 0.05^b^	21.41 ± 0.09^b^	21.24 ± 0.07^b^	21.37 ± 0.04^b^	21.14 ± 0.07^b^	20.56 ± 0.13^a^
Glycogen (mg/g)	0.3874 ± 0.0170^a^	0.4162 ± 0.0128^a^	0.4066 ± 0.0379^a^	0.4524 ± 0.0406^ab^	0.4987 ± 0.0405^ab^	0.5808 ± 0.0249^b^
**Muscle cellularity**						
Fibre diameter (μm)	42.136 ± 0.262^c^	38.043 ± 1.513^bc^	34.798 ± 1.393^ab^	31.044 ± 1.264^a^	31.796 ± 0.648^a^	35.237 ± 1.010^ab^

All data were expressed as mean ± SE. Mean values within the same row with different superscripts are significantly different (*P* < 0.05; Tukey's test).

Muscle fibre diameter was significantly affected by the dietary carbohydrate levels (*P* < 0.05) (Table 3). Dietary carbohydrates significantly decreased the muscle fibre diameter (*P* < 0.05), and the highest value was found in the C0 group.

### Ultrastructure analysis

**Figure 1:**
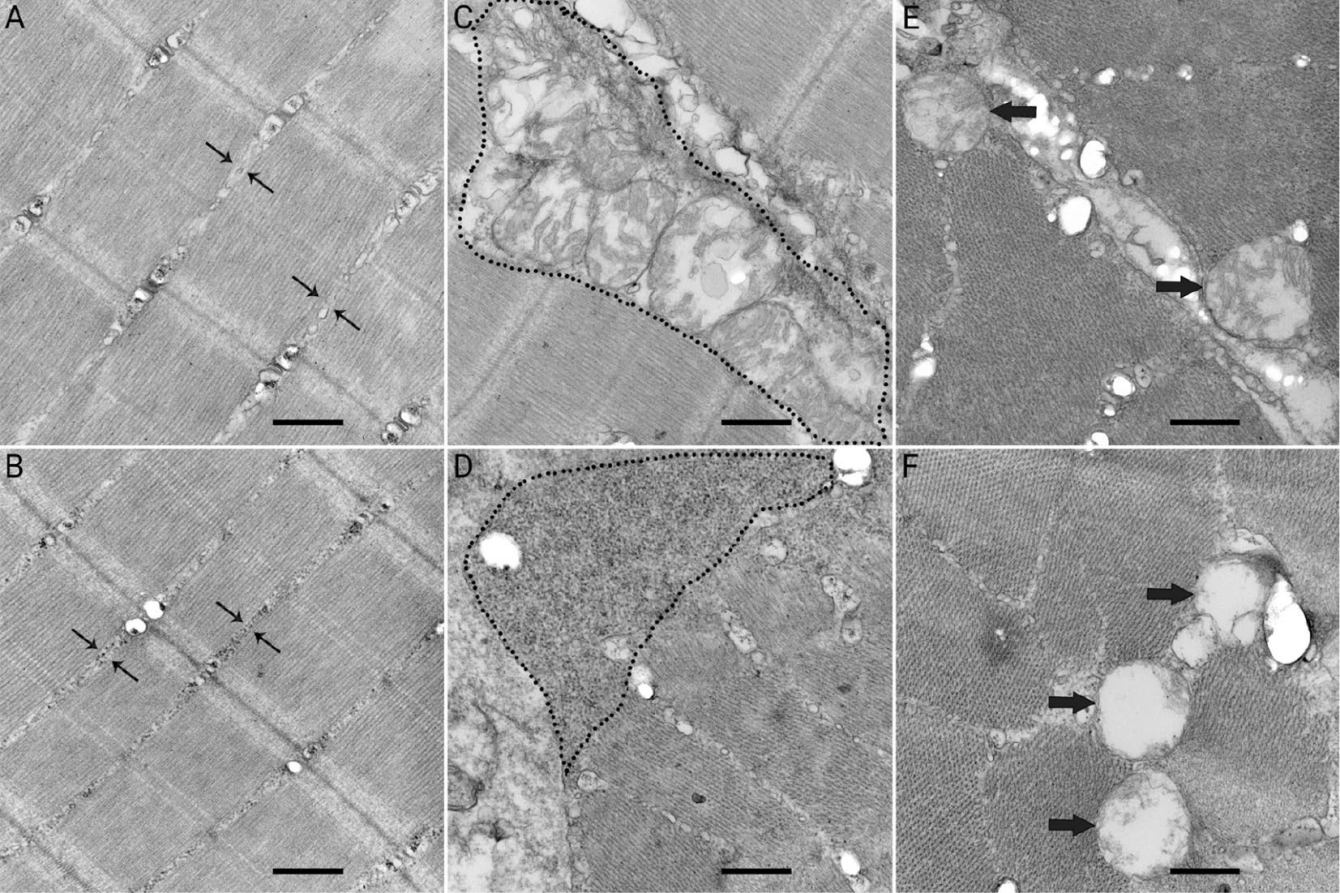
Ultra-thin section of skeletal muscle from the C0 group and the C24 group, bar = 500 nm. Little glycogen granules between the myofibrils in the C0 group (**A**). Some deposits of glycogen granules between the myofibrils in the C24 group (**B**). Mitochondria rather than glycogen granules are seen near the endomysium in the C0 group (**C**). Large accumulation of glycogen granules is seen near the endomysium of fish muscle in the C24 group (**D**). Normal mitochondrion in fish muscle in the C0 group (**E**). Swollen mitochondria and degenerated cristae were observed in the C24 group (**F**).

Observations of the transmission electron microscopy found the glycogen depositions between myofibrils (Fig. 1B) and near the endomysium (Fig. 1D) in the C24 group. Swollen mitochondria and degenerated mitochondrial cristae were also found in the C24 group (Fig. 1F). Little glycogen granules were found between the myofibrils and near the endomysium in the C0 group. No abnormalities of mitochondria were found in the C0 group.

### Protein carbonyl content, mtDNA content and AMP/ATP ratio

**Figure 2:**
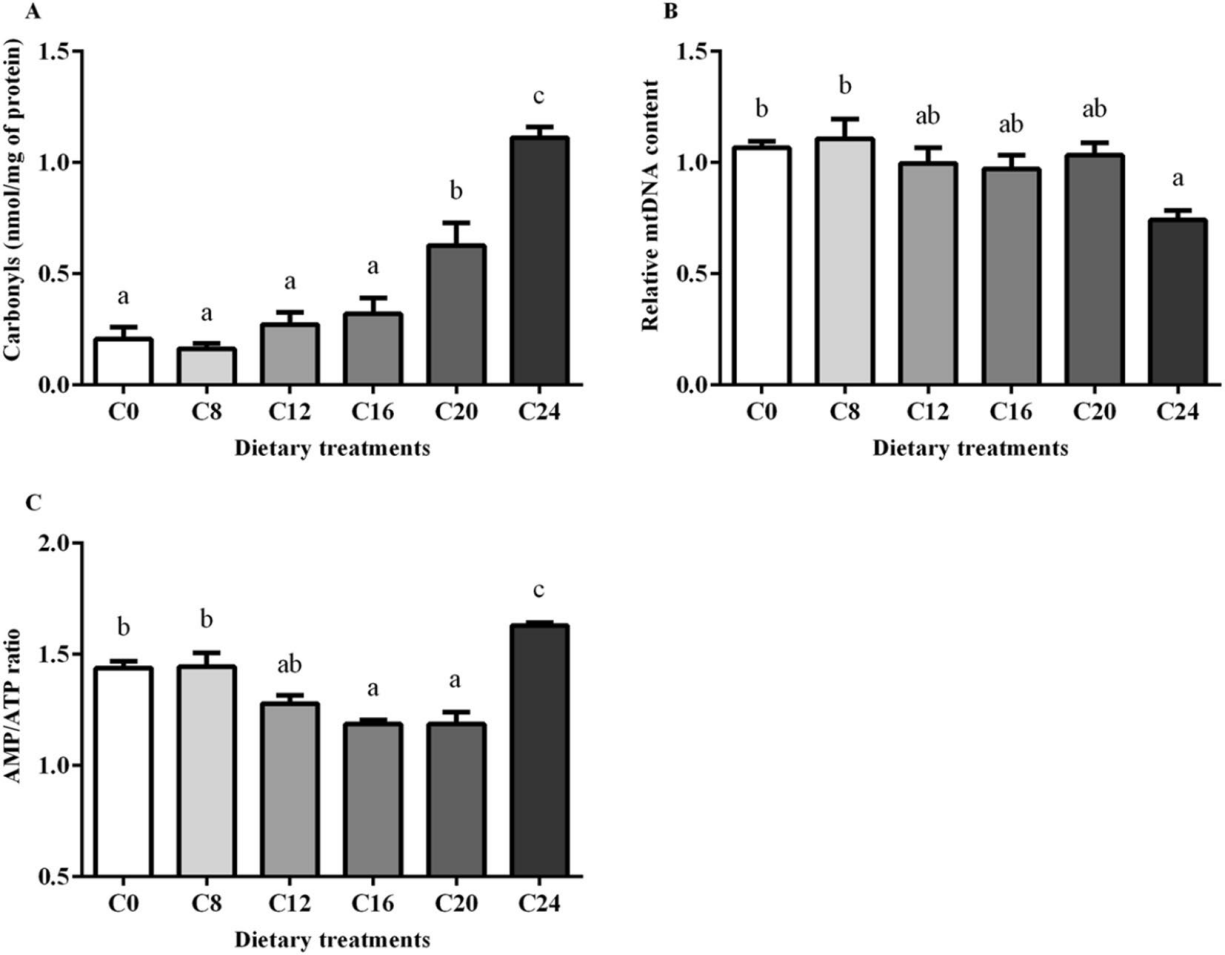
Protein carbonyl content (**A**), mtDNA content (**B**) and the AMP/ATP ratio (**C**) in skeletal muscle of olive flounder. All data were expressed as mean ± SE. Values with different letters means significant differences (P < 0.05, Tukey’s test).

Protein carbonyl contents in muscle are shown in Fig. 2A. Significantly higher contents of protein carbonyls were found in the C20 group and C24 group (*P* < 0.05). Muscle in the C24 group had the highest value of protein carbonyl content.

The mtDNA copy numbers in muscle are shown in Fig. 2B. There was a significant decrease of mtDNA copy number in the C24 group compared with that in the C0 and C8 groups (*P* < 0.05).

As the increase of dietary carbohydrate levels, the AMP/ATP ratio in muscle decreased first and then increased, and the minimum value was found in the C16 group (*P* < 0.05) (Fig. 2C). There was a significant elevation of AMP/ATP ratio in the C24 group compared with that in other groups (*P* < 0.05).

### Gene expression

**Figure 3:**
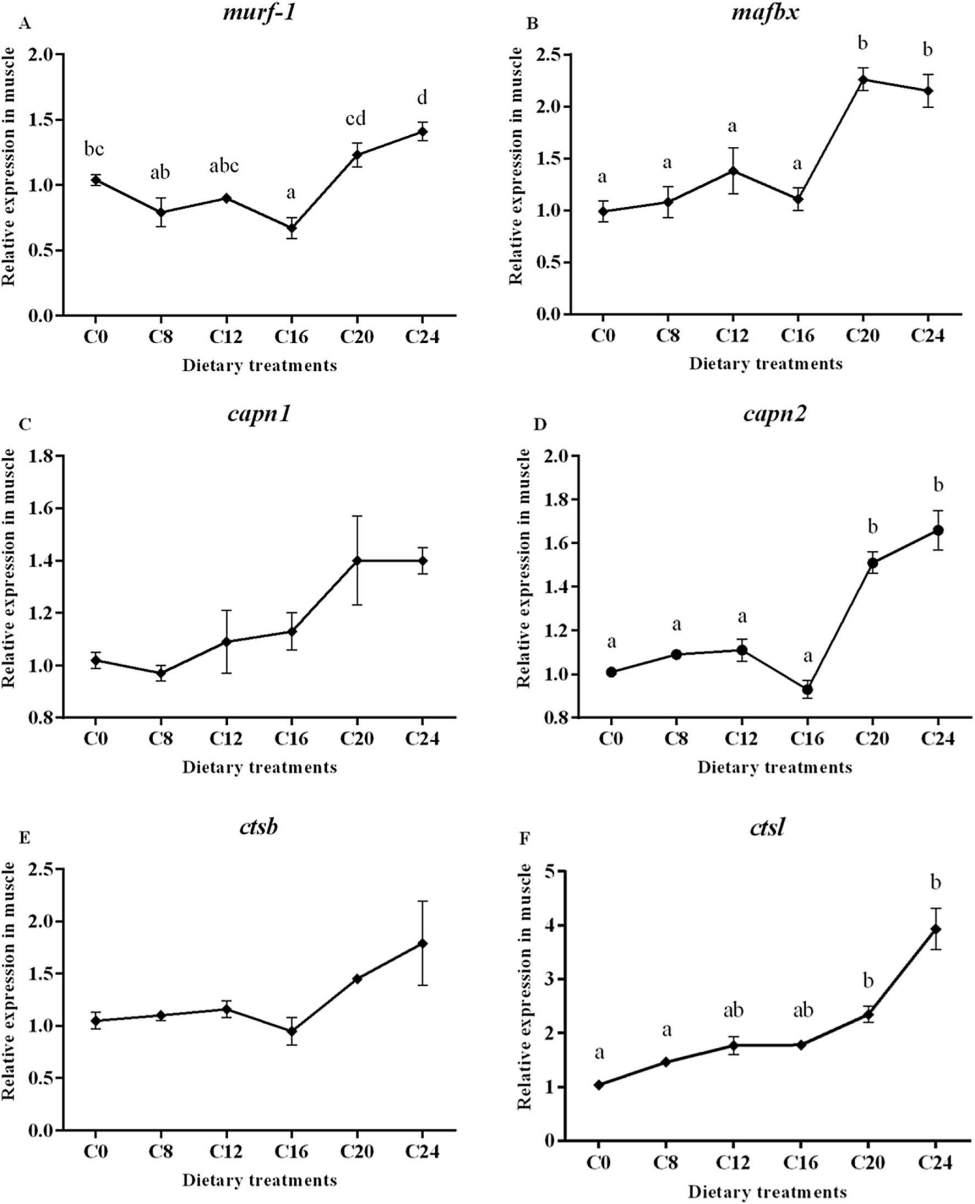

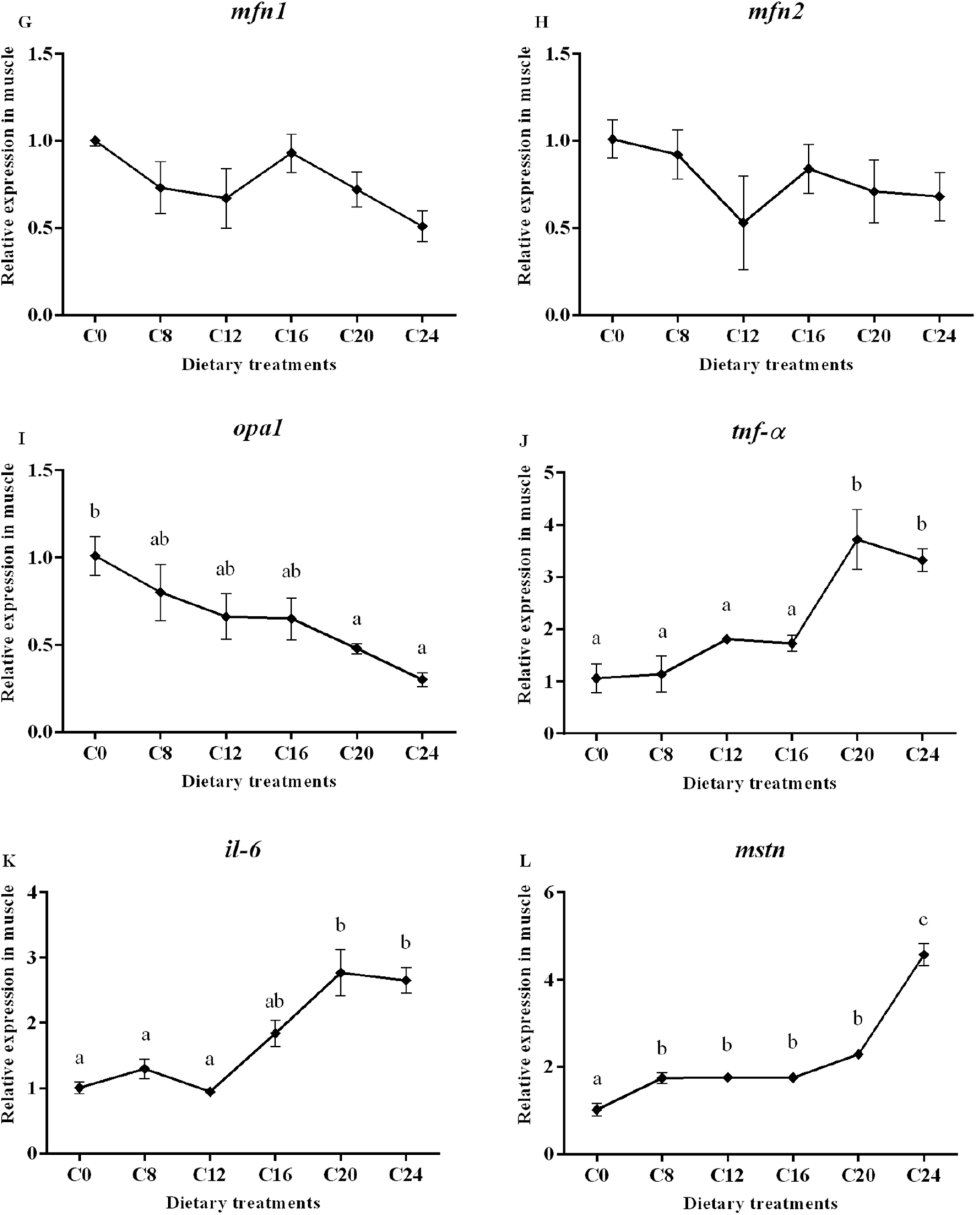

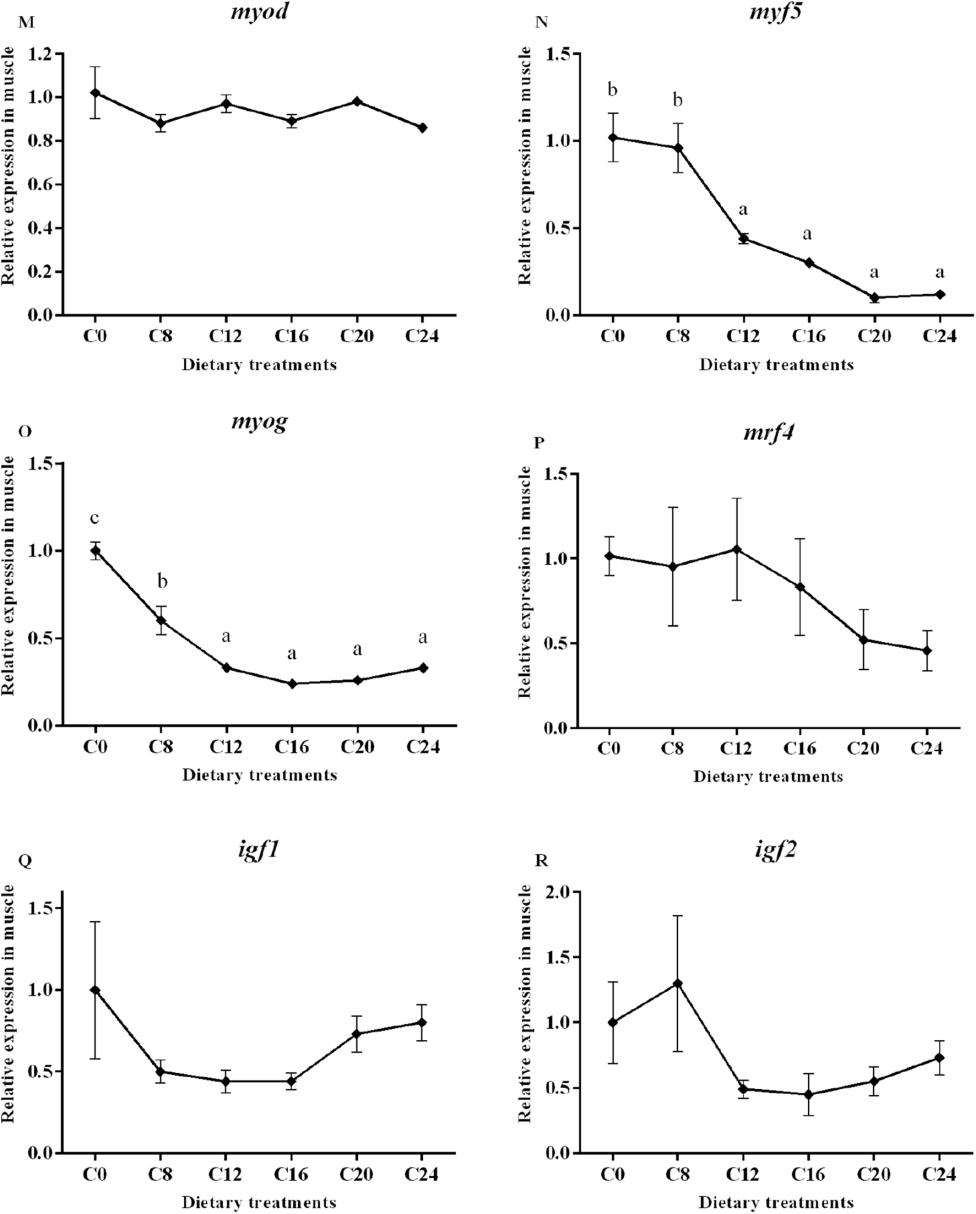

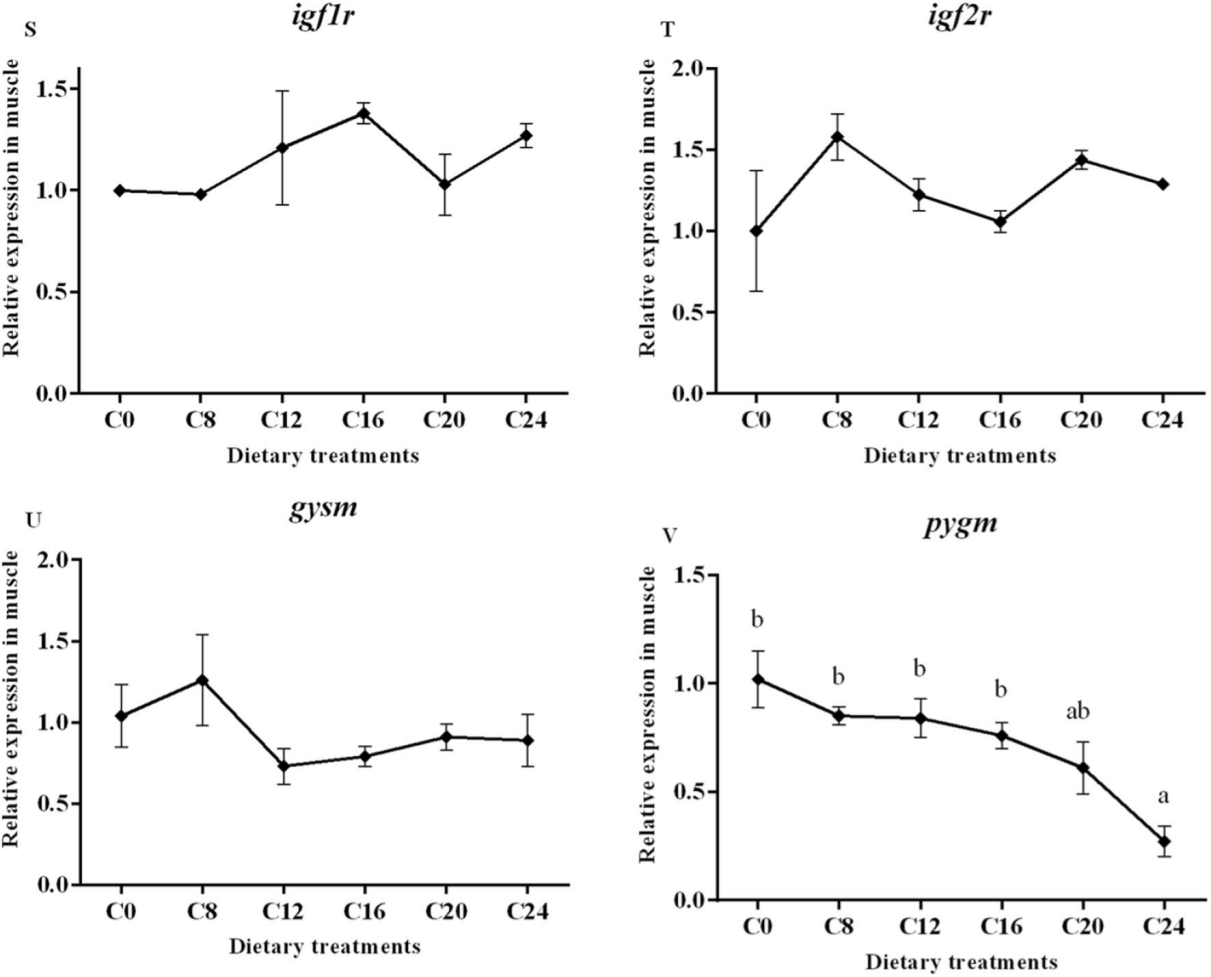
(1) Expression of genes about protein turnover (*murf-1*, *mafbx*, *capn1*, *capn2*, *ctsb* and *ctsl*), mitochondria membrane infusion (mfn1 and mfn2), cristae organization (opa1), inflammation (*tnf-α* and *il-6*), myogenic regulatory factors and mstn (*myf5*, *myod*, *myog*, *mrf4* and *mstn*), insulin-like factors receptors (*igf1r* and *igf2r*) and glycogen synthesis and glycogenolysis (*gysm* and *pygm*) in the skeletal muscle. Data are shown as mean ± SE. Values with different letters means significant differences (p < 0.05, Tukey’s test).

Data on the expression of the selected genes are presented in Fig. 3. The transcript level of muscle-specific RING finger protein 1 (*murf-1*) gene was significantly affected by dietary carbohydrate levels (*P* < 0.05), with the lowest value in the C16 group and the highest value in the C24 group. In addition, the groups of C20 and C24 showed significantly higher mRNA levels of muscle atrophy F-box protein (*mafbx*) and calpain-2 (*capn2*) compared with the other treatments (*P* < 0.05). The transcript level of cathepsinL (*ctsl*) showed a significantly increasing trend with the increase of dietary carbohydrate levels (*P* < 0.05), and the highest value was found in the C24 group. However, the expression of calpain-1 (*capn1*) and cathepsinD (*ctsd*) were not affected by dietary treatments (*P* > 0.05).

Transcript level of optic atrophy protein1 (*opa1*) was significantly decreased in the C20 and C24 groups compared with those in the C0 group (*P* < 0.05). No significant differences in mRNA levels of mitofusins 1 (*mfn1*) and mitofusins 2 (*mfn2*) were observed among all the treatments (*P* > 0.05). A significant higher transcript level of tumor necrosis factor (*tnf-α*) was detected in the C20 and C24 groups (*P* < 0.05). The transcript level of interleukin-6 (*il-6*) in the C20 and C24 groups were significantly higher than those in the other groups (*P* < 0.05).

The relative expression of *mstn* increased significantly with the increasing dietary carbohydrate levels (*P* < 0.05), and the highest value was observed in the C24 group. The gene expressions of myogenic factor 5 (*myf5*) in the C12, C16, C20 and C24 groups were significantly decreased compared with that in the C0 and C8 groups (*P* < 0.05). The mRNA level of myoblast determination protein (*myod*) and muscle-specific regulatory factor 4 (*mrf4*) showed no obvious trend (*P* > 0.05). The expression of myogenin (*myog*) decreased significantly with the increasing dietary carbohydrate levels (*P* < 0.05). Moreover, gene expression of Insulin-like growth factor I (*igf-I*)*,* Insulin-like growth factor II (*igf-II*), Insulin-like growth factor 1 receptor (*igf1r*) and Insulin-like growth factor 1 receptor (*igf2r*) were not significantly (*P* > 0.05) affected by the dietary treatments. The transcript level of glycogen synthase (*gysm*: muscle type) was not significantly (*P* > 0.05) affected by dietary treatments, while the gene expression of glycogen phosphorylase (*pygm*: muscle type) was significantly lower in the C24 group than that in the C0, C8, C12 and C16 groups.

### Western blot

**Figure 4:**
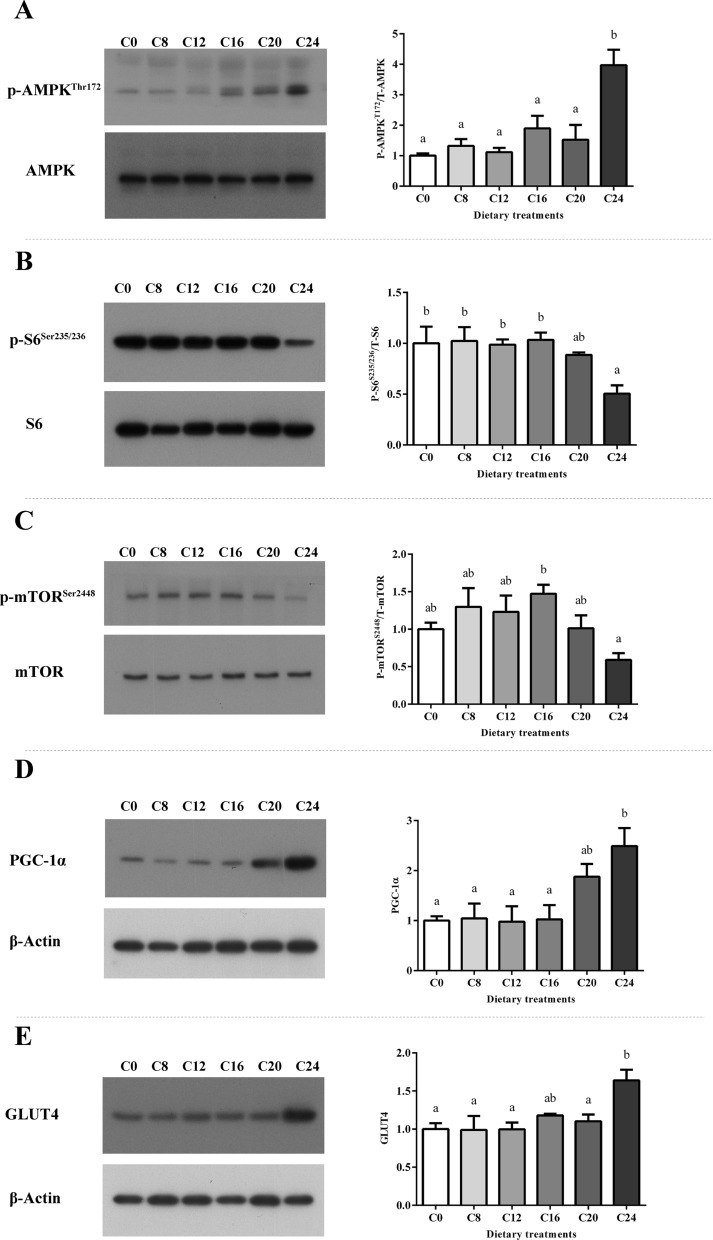
The levels and phosphorylation of AMPK (**A**), S6 (**B**) and mTOR (**C**), and the levels of PGC-1α (**D**) and GLUT4 (**E**) were examined by western blots and quantitated. Results are represented as mean ± SE. Values with different letters means significant differences (*P* < 0.05, Tukey’s test).

Significant increase of phosphorylation of AMPK (Thr^172^) was observed in the C24 group. The phosphorylation level of S6 (Ser^235/236^) was significantly decreased in the C24 group (*P* < 0.05). The phosphorylation level of mTOR (Ser^2448^) in the C16 group was significantly higher than that in the C24 group (*P* < 0.05). Upregulation of PGC-1α level was found in the C24 group compared with the C0, C8, C12 and C16 groups, while upregulation of GLUT4 level was found in the C24 group compared with the C0, C8, C12 and C20 groups (Fig. 4, *P* < 0.05).

## Discussion

Muscle pH of fish in post-mortem is a crucial quality parameter^36–38^. A lower post-mortem muscle pH affects the texture, water holding capacity and the proteolytic activity in fish^38,39^. Previous studies in Atlantic salmon (*Salmo salar*)^40,41^ and cod (*Gadus morhua*)^42^ suggested that the glycogen level in muscle is the principal determinant of post-mortem pH due to its anaerobic glycolysis to lactic acid^43^. Significantly lower muscle pH in the C20 and C24 groups was observed, which suggested higher lactate level post-mortem. In fish, dietary carbohydrate may increase the glycogen in muscle. It was reported in dentex (*Dentex dentex*)^10^ that muscle glycogen content is higher in fish fed diets with a high level (28%) of carbohydrate. In present study, the glycogen content in fish muscle of the C24 group was 0.5808 mg/g, which is significantly higher than that in the C0, C8 and C12 groups. Higher muscle glycogen caused by dietary carbohydrate increased the glycolytic substrate and might ultimately decrease the muscle pH.

The LHC is an important flesh quality attribute in fish^44^. It is typically evaluated by water loss, lipid loss and liquid loss. In present study, high dietary carbohydrate level (24%) led to the significant increases of all these three parameters. Changes in muscle microstructure and acidification from anaerobic glycolysis are two critical factors for the LHC^36,45,46^. Compared with that in the C0 group, in present study, the changes of muscle ultrastructure (glycogen accumulation both between the myofibrils and near the endomysium) in the C24 group were observed (Fig. 1). This could be part of the reason for the increase the water loss and lipid loss of muscle in olive flounder. Meanwhile, the muscle pH was decreased in the C24 group. A decreased muscle pH increased the liquid loss of fish muscle by causing muscle swelling^47,48^, so the variation in muscle pH also partly contributed to the change of LHC in olive flounder.

Fillet texture is one of the most important quality parameters in fish. The textural mechanical properties (hardness, springiness, chewiness, cohesiveness and adhesiveness) are wildly used to evaluate muscle texture. In present study, the hardness, springiness, chewiness and adhesiveness significantly decreased with increasing dietary carbohydrate levels. A similar result was reported in previous study, in which it was showed that diet with high carbohydrate content (28%) resulted in lower value of muscle hardness in dentex^10^.

Muscle fibre diameter is known to modify the textural properties of fish muscle. Some previous studies found that there is a negative correlation between fillet hardness and muscle fibre diameter in Atlantic salmon, sea bass (*Dicentrarchus labrax*) and gilthead sea bream (*Sparus aurata*)^37,49,50^. However, in preset study, dietary carbohydrate decreased the muscle fibre diameter as well as the muscle hardness in olive flounder. It is consistent with those findings in Senegalese sole (*Solea senegalensis*)^51^. Other factors of the muscle structure can counterbalance the contribution of muscle fibre to texture^52^. Previous studies showed that a lower pH is often associated with post-mortem textural modifications^47,53^, because a lower muscle pH may reduce connective tissue strength and cause softer flesh^54^. At the same time, change in ultrastructure is associated with muscle texture in fish^55,56^. In present study, transmission electron microscopy (TEM) technique was used to analysis the possible difference of muscle ultrastructure between fish in the C0 and C24 group. Glycogen accumulations between myofibrils, large glycogen aggregates near the endomysium and abnormal mitochondria were observed in the C24 group with softer texture muscle. Similarly, TEM investigations of soft and hard muscle in Atlantic salmon found that the soft flesh was related to the massive intracellular glycogen accumulation associated with degenerated mitochondria^56^. Endomysium and fibre detachment caused the loss of fillet hardness in Atlantic salmon^55^. In present study, large accumulation of glycogen granule near the endomysium influenced the endomysium-fibre attachment in muscle in the C24 group, and thus reduced the muscle hardness. It is indicated that high glycogen content and lower pH in muscle might reduce the muscle hardness of olive flounder.

Cathepsin B and L are lysosomal proteases, and calpains are cytosolic proteases. They involve in the breakdown of muscle structure and leading to the muscle tenderization in fish^22,57–59^. The action of endogenous protease can modify muscle properties^54,60^. Expressions of *cathepsin* and *calpain* were related to the muscle texture, and could be regulated by diet composition^15,61^. In present study, expressions of calpain 2 and cathepsin L were significantly upregulated with the increasing of dietary carbohydrate levels. According to Bahuaud et al., cathepsin L rather than cathepsin B is related to the fillet hardness^12^. Calpains and cathepsins act synergistically to dissociate and degrade myofibrillar protein in the early post-mortem period^62^. Ubiquitin protease (Ubp) system is an important proteolytic pathway involved in fish muscle atrophy^63^. Among the Ubp system members, the muscle RING-finger Protein 1 (MuRF-1) and the muscle atrophy F-box Protein (MAFbx) are key E3 ubiquitin ligases specifically expressed in muscle^64^. A significantly negative correlation between muscle hardness and expressions of the Ubp related genes was found in rainbow trout (*Oncorhynchus mykiss*)^65^. Elevated protein degradation in fish muscle was associated with the decreased fish flesh hardness^65^. Present work showed that dietary carbohydrate level significantly influenced the expression of two Ubp related genes (*murf-1* and *mafbx*). The increased expressions of *calpain 2*, *cathepsin L*, *murf-1* and *mafbx* suggested the elevated protein degradation in the C24 group. These results indicate that excessive dietary carbohydrate activated the proteolysis process thus reduced the hardness of muscle in olive flounder.

Reactive oxygen species (ROS) generated in the process leading to oxidative stress is responsible for the formation of protein carbonyl^66,67^. It was found, in present study, that protein carbonyl in muscle was significantly increased with the increasing dietary carbohydrate levels. Higher content of protein carbonyl in the muscle of fish in the C20 and C24 groups indicated the higher oxidative stress in these two groups. As key components of the stress response, mitochondria may change their function on biogenesis, metabolism, ROS generation and apoptosis during the state of stress^68^. At the same time, mitochondria also act as the most immediate targets of the oxidative damage inflicted by ROS^69,70^. As mtDNA lacks histone proteins, and the generation of ROS in mitochondria, mtDNA is more sensitive to oxidative damage comparing to nuclear DNA^71^. The mitofusins, Mfn1 and Mfn2 participate in fusion of the outer mitochondrial membrane^72^. OPA1 is a mitochondrial protein, and plays a fundamental role in the fusion of the inner membrane, organization of mitochondrial cristae and apoptosis^73^. The mtDNA copy number is considered as a marker of mitochondrial biogenesis^74^ and energetic function^75^. The present study found that the mtDNA copy number significantly decreased in the C24 group. Meanwhile, the gene expression of *opa1* was decreased with increasing dietary carbohydrate levels. It was suggested that the biogenesis, health and function of mitochondria in olive flounder were influenced by higher dietary carbohydrate contents. It was also confirmed by swollen mitochondria and degenerated cristae in the C24 group showed by the TEM result (Fig. 1F).

The muscle growth in fish consists of the recruitment of new muscle fibres (hyperplasia) and the enlargement of existing muscle fibres (hypertrophy). These two processes need the satellite cell proliferation and differentiation^76^. The MSTN is regarded as a negative regulator of muscle growth in teleost fish^77–79^. It inhibits cell proliferation as well as differentiation during myogenesis in zebrafish (*Danio rerio*)^80^. It also negatively regulates MRFs expression, thus inhibits myofibrogenesis. Researches in zebrafish and spotted rose snapper (*Lutjanus guttatus*) found that the knock-down of *mstn* upregulated the expression of muscle specific transcription factors^80,81^. The myogenic regulatory genes encode a family of transcription factors including MyoD, Myf5, MyoG and MRF4. Myod and Myf5 determine the muscular lineage, while MRF4 and MyoG are factors involved in cell specification and differentiation^17,82^. The present study found that gene expression of *mstn* in muscle of olive flounder was significantly up-regulated by the increase of dietary carbohydrates levels. At the same time, *myf5* and *myog* expressions were significantly downregulated by dietary carbohydrates. The lower mRNA levels of *myf5* and *myog* reflected a decreased satellite cell activity, which suggested reductions in muscle hyperplasia and hypertrophy. In rainbow trout, it was also found that excessive dietary carbohydrates (35%) down-regulated the MRFs^83^. The downregulation of MRFs is occurred simultaneously with the upregulation of *mstn*, which indicates that MSTN may also have a negative impact on the transcription of MRFs genes in olive flounder.

Muscle protein deposition is a result of both protein synthesis and protein degradation^19^. The upregulation of *mstn* could activate protein degradation via Ubp system. Research in spotted rose snapper^81^ found that *mstn* gene silencing could restrained the transcriptional of *murf-1*, and MSTN has been reported to activate Ubp system members (Murf-1 and MAFbx)^84^. That could be confirmed in present study since muscle crude protein significantly decreased in the C24 group, and expressions of Ubp related genes (*murf-1* and *mafbx*) were significantly increased with the increase of *mstn* transcription. Insulin-like growth factor (IGF) system is a major hormone axis regulating protein synthesis and cellular dynamics of muscle growth^85,86^. Banos et al. reported that the number of IGF-I receptors could be upregulated by carbohydrate enriched diet in rainbow trout^87^, but that could not be confirmed in present study. The expressions of *igf-I*, *igf-II*, *igf1r* and *igf2r* remained unaffected by dietary carbohydrate levels. Moreover, TORC1/p70S6k signaling pathway is crucial to the protein synthesis and cell growth^88,89^. It is reported that S6 is a primary substrate of p70S6K and its phosphorylation level reflects the phosphorylation level of p70S6K. Previous study found that MSTN can decrease the activity of the TORC1/p70S6k signaling pathway^90^. In present study, the phosphorylation level of S6 (Ser235/236) was significantly decreased in the C24 group. Decreased phosphorylation level of S6 suggested the suppression of protein synthesis in the C24 group.

The AMPK acts as a key sensor of fuel and energy status in skeletal muscle that mediates the cellular adaptation to environment or nutritional stress factors^91^. It is activated by the increase of intracellular AMP/ATP ratio^92^. As a basic energy substance, carbohydrate plays an important role in the energy metabolism. However, with the increase of dietary carbohydrate levels, the AMP/ATP ratio showed a trend of first decreasing and then increasing in present study. An ‘energy deficiency’ state in skeletal muscle and the increased phosphorylation of AMPK (Thr 172) was observed in the C24 group. The decreasing trend might be attributed to the increase of gross energy as the addition of carbohydrate in diet. While the significantly higher AMP/ATP ratio in the C24 group could in part as a result of disturbed energy homeostasis caused by the changes in mitochondrial health and biosynthesis. Results in present study demonstrated that excessive dietary carbohydrate (24%) modulated the AMPK/mTOR/S6 pathway, evidenced by the increased phosphorylation of AMPK and reduced phosphorylation level of S6 (Fig. 4). The phosphorylation of S6 is positively associated with regulating cell size, glucose homeostasis and protein synthesis^93^. S6 takes part in binding mRNA, and its phosphorylation has a regulatory role in translation initiation^94^. The mTOR is a protein serine/threonine kinase activated by growth factors and nutrient rich conditions^95^. Activated mTOR positively regulates protein synthesis and skeletal muscle mass through direct phosphorylation of downstream proteins, p70S6K and 4E-BP1^21^. Ser2448 has been proved to be the phosphorylation site of AKT in mTOR^96–98^. It has been demonstrated that down regulation of AKT/mTOR signaling promotes autophagy and protein degradation in skeletal muscle^99^. In present study, phosphorylation (Ser^2448^) level of mTOR in the C24 group was significantly lower than that in the C16 group, which is in coincidence with the specific growth rates of these two dietary carbohydrate contents^33^.

It is noteworthy that the protein level of PGC-1α was increased in the C24 group. Previous studies in fish and mammals reported that the activation of AMPK increased the mRNA and protein levels of PGC-1α^100,101^. Several studies demonstrated that ROS can also increase the expression of PGC-1α^102,103^. In present study, AMPK was activated in the C24 group. Meanwhile, the increased ROS proved by the elevated content of Protein carbonyl was found as the increasing of dietary carbohydrate levels. These two factors positively regulated the protein level of PGC-1α in C24. It is reported that upregulation of PGC-1α enhances intramuscular glycogen storage via increasing basal glucose transport and the downregulation of glycogen phosphorylase^104–106^. GLUT4, highly expressing in adipose tissue and skeletal muscle, plays an important role in glucose uptake^107^. As a downstream factor positive regulated by PGC-1α^108^, GLUT4 showed a similar expression trend with PGC-1α in present study, which showed that the glucose uptake in skeletal muscle was increased under 24% dietary carbohydrate condition. It is proved that in brown trout (*Salmo trutta*) skeletal muscle cells, the activation of AMPK increase glucose uptake through a GLUT4-mediated mechanism by increasing the cell surface and mRNA level of GLUT4^101^. Glycogen synthase and glycogen phosphorylase are the key enzymes in glycogen synthesis and glycogenolysis respectively. Glycogen phosphorylase promotes the use of glycogen as an energy source and is downregulated by PGC-1α. Gene expression of *pygm* was significantly downregulated in the C24 group, while there was no significant difference of the expression of *gysm* among all the groups, which would decrease the glycogenolysis in skeletal muscle in the C24 group. The result of TEM (glycogen accumulation) combined with significantly higher muscle glycogen content showed an impaired muscle glycogen metabolism in the C24 group. The upregulation of PGC-1α and GLUT4 activated by AMPK in the C24 group increase the glucose transferred across the plasma membrane, while the downregulation of *pygm* transcription decreased the utilization of glycogen for energy generation. This imbalance between glucose uptake and utilization caused glycogen accumulation, on the other hand influenced the energy homeostasis.

Bentonite is natural clay that comes from volcanic ash^109^. It has the properties of safety, improved flow ability, good pellet quality and anti-caking. Because of properties and accessibility, bentonite is commonly used as a feed additive^110^. Bentonite was used as inert filler in some researches of fish nutrition. Diet formulation was adjusted by manipulating the bentonite content^111–113^. However, some studies showed that the bentonite in feed can alleviate toxicity (e.g., induced by dietary aflatoxin B1, plumbum and cadmium) by decreasing toxic substances residues in fish bodies, rehabilitating the enzyme activity and modifying the function of kidney and liver in fish^114–117^. These results might explain the reported beneficial effects of feeding bentonite to fish in some cases^118,119^. To the best of our knowledge, there is no research on the influence of dietary bentonite on the muscle texture parameters. There were no grade levels of bentonite in the diets’ formulation in the present study either. So, it is difficult to conclude whether bentonite has impact on the muscle texture of olive flounder or not in the present study. Further studies are needed.

## Conclusion

**Figure 5:**
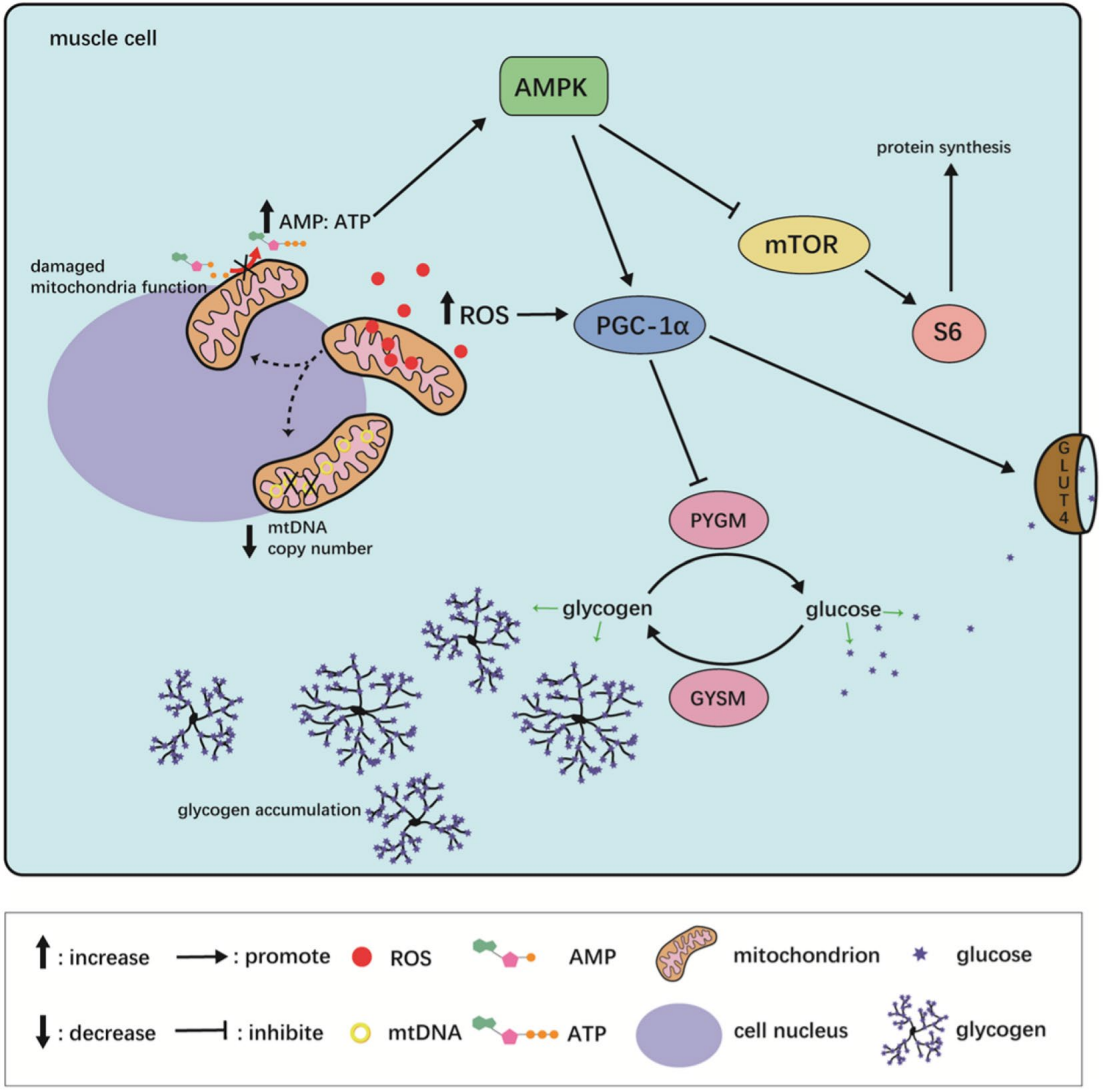
The summarized mechanism of the effect of high dietary carbohydrate level on muscle quality of olive flounder.

In conclusion, excessive dietary carbohydrate level (24%) caused oxidative stress, upregulation of *mstn*, damage of mitochondria function and biosynthesis. It influenced the energy homeostasis and the activity of AMPK. The activated AMPK subsequently inhibited S6, which is the downstream effector of mTOR. At the same time, activated AMPK improved the protein level of PGC-1α and GLUT4, which ultimately enhanced intramuscular glycogen storage. The AKT/mTOR signaling was also inhibited by excessive dietary carbohydrate content. These effects subsequently suppressed protein synthesis while promote protein degradation, affected myogenesis along with cellularity and led to the accumulation of muscle glycogen, which in turn influenced muscle quality of olive flounder (Fig. 5).

## Materials and methods

### Ethical statement

The present study was performed in strict accordance with the recommendations in the Guide for the Use of Experimental Animals of Ocean University of China. The protocols for animal care and handing used in this study were approved by the Institutional Animal Care and Use Committee of Ocean University of China.

### Experimental diets

Six isonitrogenous and isolipidic graded levels of carbohydrate (0%, 8%, 12%, 16%, 20% and 24%, respectively) were designed. The α-starch and corn starch were used as the carbohydrate sources. All ingredients were finely ground, well mixed, and dry extruded in a laboratory pellet mill (EL220, Shandong Haiyang, China). The diameters of the diets were 3 mm and 5 mm. The sinking pellet diets were dried in a forced air oven at 50 °C for 8 h and stored in a refrigerator (− 20 °C) until used. The diets were named as C0, C8, C12, C16, C20 and C24, respectively. The analyzed dietary carbohydrate contents were 0.93, 7.01, 11.06, 15.60, 19.96 and 23.18% (dry matter), respectively (Table 4).

**Table 4:** Formulation and proximate compositions of the experimental diets (% of dry matter).

Ingredients	Diets					
	C0	C8	C12	C16	C20	C24
Fish meal	57	57	57	57	57	57
Wheat gluten	13	13	13	13	13	13
Alpha-starch	0	5	5	5	5	5
Corn starch	0	1.7	5.7	9.7	13.7	17.7
Soybean lecithin	1	1	1	1	1	1
Fish oil	3.5	3.5	3.5	3.5	3.5	3.5
Choline chloride	0.4	0.4	0.4	0.4	0.4	0.4
Ethoxyquin	0.05	0.05	0.05	0.05	0.05	0.05
Mold inhibitor^a^	0.1	0.1	0.1	0.1	0.1	0.1
Monocalcium phosphate	0.5	0.5	0.5	0.5	0.5	0.5
Vitamin premix^b^	0.6	0.6	0.6	0.6	0.6	0.6
Minerals premix^c^	0.5	0.5	0.5	0.5	0.5	0.5
Y_2_O_3_	0.1	0.1	0.1	0.1	0.1	0.1
Bentonite	6.7	0	0	0	0	0
Carboxymethyl cellulose	16.55	16.55	12.55	8.55	4.55	0.55
Total	100	100	100	100	100	100
**Proximate analysis (% dry matter)**						
Dry matter (% diet)	95.65	96.80	96.61	97.23	97.08	96.96
Crude protein	49.96	49.59	49.49	49.60	49.99	50.44
Crude lipid	9.59	9.73	9.65	9.98	9.75	9.96
Carbohydrate^d^	0.93	7.01	11.06	15.60	19.96	23.18
Ash	17.73	12.46	11.72	11.73	11.93	12.13

^a^Mold inhibitor: 50% calcium propionic acid and 50% fumaric acid.

^b^Vitamin premix (g kg^−1^ of diet): microcrystalline cellulose, 9.884 × 10^–2^; VA, 1.92 × 10^–4^; VB1, 1.5 × 10^–4^; VB2, 2.7 × 10^–4^; VB6, 1.2 × 10^–4^; VB12, 6 × 10^–5^; VD, 2.1 × 10^–4^; VE, 1.44 × 10^–3^; VK, 6 × 10^–5^; calcium pantothenate, 3.6 × 10^–4^; nicotinic acid, 1.2 × 10^–3^; folic acid, 1.2 × 10^–4^; biotin, 3.6 × 10^–4^; inositol, 4.8 × 10^–3^; VC phosphate, 1.2 × 10^–2^.

^c^Mineral premix (g kg^−1^ of diet): MgSO_4_·7H_2_O, 6 × 10^–3^; CuSO_4_·5H_2_O, 5 × 10^–5^; FeSO_4_·H_2_O, 4 × 10^–4^; ZnSO_4_·H_2_O, 2.5 × 10^–4^; MnSO_4_·H_2_O, 2.25 × 10^–4^; CoCl_2_·6H_2_O (1%), 2.5 × 10^–4^; Na_2_SeO_3_ (1%), 1 × 10^–4^; calcium iodate, 3 × 10^–4^; zeolite powder, 4.243 × 10^–2^.

^d^Determined by the 3,5-dinitro salicylic acid method^120^.

### Feeding trial

Olive flounder juveniles (initial body weight: 7.14 ± 0.10 g) were purchased from a commercial fish farm in Haiyang (Shandong, China). Before the feeding trial, the fish were reared in tanks (3000 L) and fed the C0 diet for two weeks to acclimate to the experimental conditions. After that, fish were fasted for 24 h, weighed and assigned randomly to 18 tanks (3000 L, 150 fish per tank) with a re-circulating water system. Fish were hand-fed to apparent satiation twice daily (8:00 and 18:00). During the 10-week feeding trial, the water temperature ranged from 21 to 24 ℃, dissolved oxygen was higher than 7.4 mg/L, and salinity ranged from 30 to 33.

### Sample collection

At the end of the feeding trial, fish were not fed for 24 h. After that, fish were anaesthetized with MS-222 (50 mg/L) (Sigma, USA) and killed by a sharp blow to the head. Dorso-lateral muscle of the eye side from three fish per tank were excised and kept on ice for texture, pH and LHC analysis in 24 h. Another three fish from each tank were manually filleted, the eye side muscle for muscle composition were stored at -80℃ until analysis. Muscle samples (9 fish per treatment) were collected from the dorsal region, fixed in 10% (v/v) neutral buffered formalin for 24 h and transferred to 70% ethanol for histological analysis. At the same time, the muscle for protein carbonyl content, mtDNA content, AMP/ATP ratio analysis, gene expression and western blot analysis were collected (9 fish per treatment), transferred into RNAase-free tubes (Axygen, USA), frozen quickly in liquid nitrogen and stored at -80℃. Blocks of muscle (1 mm^3^) (9 fish per treatment) from the C0 and C24 group were carefully cut, fixed in 2.5% glutaraldehyde phosphate buffered saline for ultrastructure observation.

### Muscle pH, LHC and texture analysis

Muscle pH was determined using a pH meter (Testo 205, Testo, Germany) by inserting the glass electrode into the fillets.

The LHC was measured by gravimetric method^121^. About one gram of skinned muscle was weighed (S) and wrapped in the filter paper (V1), then centrifuged at 500×*g* for 10 min at 10 ℃. The wet filter paper (V2) was weighed and dried in 75℃ to constant weight (V3). The following parameters were calculated as:

$$ {\text{Liquid loss }} = {1}00 \, \times \, \left( {{\text{V2}} - {\text{V1}}} \right) \, /{\text{ S}} $$

$$ {\text{Water loss }} = { 1}00 \, \times \, \left( {{\text{V2}} - {\text{V3}}} \right) \, /{\text{ S}} $$

$$ {\text{Lipid loss }} = { 1}00 \, \times \, \left( {{\text{V3}} - {\text{V1}}} \right) \, /{\text{ S}} $$

**Figure 6:**
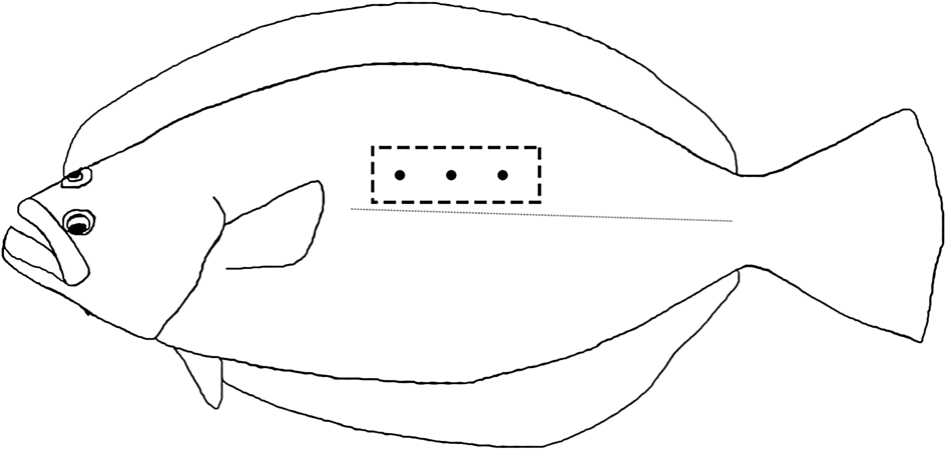
Sample sites in muscle for instrumental texture measurement. Three sampling points were detected in epaxial muscle (between dorsal and tail, above the lateral line) for each fillet.

The texture profile analysis (TPA; double compression) test was performed instrumentally using a texture analyzer (TMS-PRO, FTC, America) equipped with 8 mm cylinder probe. Three sampling points were detected in epaxial muscle (between pectoral fin and tail, above the lateral line) for each fillet (Fig. 6). The probe moved downward at a constant speed of 30 mm/min, the initial force was 0.1 N with 60% deformation of the original length^122^. The equipment measured the hardness, cohesiveness, springiness, adhesiveness and chewiness of the fillets.

### Muscle composition analysis

The proximate composition of muscle was analyzed according to the standard methods (AOAC, 1995). Crude protein was determined by Kjeldahl method (2300, FOSS, Sweden) by measuring nitrogen (N × 6.25). Crude lipid was measured after diethyl ether extraction by Soxhlet method (Extraction System-811, BUCHI, Switzerland).

Glycogen content in muscle was determined using the commercial kit (Nanjing Jiancheng Bioengineering Institute, Nanjing, China) according to the manufacturer’s instructions. The absorbance was measured at 620 nm with a spectrophotometer (UV-2401PC, Shimadzu, Japan). Glycogen contents were expressed as mg per g of muscle (wet weight).

### Histological analysis

Samples were dehydrated by grade ethanol for 10 min in each gradient concentration (70%, 80%, 85%, 90%, 95% and 100%). After dehydration, samples were embedded in paraffin wax (PPDT-12C1, Ceike, China). The tissue blocks were sectioned at 7 μm thickness using a rotary microtome (RM2235, Leica, Germany). The sections were then placed on the slides and stained with hematoxylin and eosin (H&E, Nanjing Jiancheng Bioengineering Institute, Nanjing China) using the slide stainer (Thermo Gemini A2, USA). Slides were viewed under light microscopy. And images were acquired using a digital camera (Olympus DP72, Nikon, Japan), and then analyzed using Image-Pro Plus 6.0 software. Mean individual muscle fibre area (${\overline{\text{a}}}$, μm^2^) was determined based on the methodology used by Valente et al.^51^. More than 900 muscle fibre sections in one sample were measured by circumscribing the physical limits of fibres. Muscle fibre diameter was computed as follows: D (muscle) = 2${\overline{\text{a}}}$^0.5^ π^−0.5^.

### Transmission electron microscopy

The glutaraldehyde-fixed tissues were washed with 0.1 M phosphate buffer saline (PBS) and placed in 1% OsO4 for post-fixation and dehydrated in ascending acetone series. After dehydration, the specimens were infiltrated and embedded in different concentrations of Epoxy resin (EPON 812, TAAB Laboratories Equipment Ltd, UK) (acetone: resin ratio of 2:1 for 0.5 h in room temperature, acetone: resin ratio of 1:2 for 1.5 h in 37 ℃ and 100% acetone for 2 h in 37 ℃). Ultra-thin (70 nm) sections were obtained using an ultramicrotome (Reichert Jung Ultracut E, Austria) and mounted on uncoated copper grids. The sections were then stained with uranyl acetate and lead citrate and examined by transmission electron microscope JEM 1200 (JOEL, Japan).

### Protein carbonyl content

Approximately 30 mg of muscle and 200 μL radioimmunoprecipitation lysis buffer (cat. No. R0020, Beijing Solarbio Science and Technology Co., Ltd., Beijing, China) were added to the centrifuge tube and homogenized using a motor-driven tissue grinder (cat. No. G506003, Sango, China). The homogenate was centrifuged and the supernatant was tested using protein carbonyl assay kit (cat. No. A087, Nanjing Jiancheng Bioengineering Institute, Nanjing, China). The absorbance values at 315 nm were determined on a microplate reader (Spectra Max i3x, Molecular Devices, USA). The protein concentrations were determined using a Bicinchoninic Acid Protein assay kit (cat. No. P0009, Beyotime Institute of Biotechnology, Shanghai, China). The carbonyl content was described as nmol per mg protein.

### Determination of mtDNA content

Total DNA from the muscle was isolated using the QIAamp DNA Mini kit (Qiagen, Germany) according to the manufacturer’s instructions. qPCR experiments were carried out with an ABI7500 RT-PCR system (Applied Biosystems, USA) using TB Green Fast qPCR Mix (TaKaRa, Japan). Each measurement was carried out three times, using 10 ng of DNA. The data were analyzed by the ΔΔCt method. And mtDNA content was calculated based on measuring the amount of NADH dehydrogenase, subunit 1 (forward primer 5′-CCTCACAGGGGTTCACTC-3′; reverse primer 5′-GTCCTCCTGCATACTCGACG-3′) relative to that of nuclear DNA encoded β-actin gene (forward primer 5′-GGAAATCGTGCGTGACATTAAG-3′ and the reverse primer 5′-CCTCTGGACAACGGAACCTCT-3′).

### AMP/ATP ratio analysis

The AMP/ATP ratio analysis was performed as described by Ryder^123^ with some modifications. Briefly, muscle sample was homogenized in cold 0.6 M HCLO_4_ and centrifuged to get the supernatant. Then, 10 ml of supernatant was incubated at 0 ℃ for 30 min and filtered. After that, the samples were diluted with potassium phosphate buffer to 20 ml and kept at – 80 ℃ before analysis. Adenosine 5′-monophosphate (AMP) disodium salt (cat. No. 01930, Sigma Aldrich, USA) and Adenosine 5′-triphosphate (ATP) disodium salt hydrate (cat. No. A7699, Sigma Aldrich, USA) were diluted with phosphate buffer to working solutions to make the standard curve. Analysis was carried out using a HPLC system (HP1100, Agilent Technologies, USA) in the conditions as follow: sample injection, 5 μl; flowrate, 1.0 ml/min; wave length, 260 nm.

### Gene expression

RNA from muscle was extracted by Trizol Reagent (Invitrogen, USA) and quantified on a spectrophotometer (Nanodrop2000, USA). Reverse transcription was performed using PrimeScript RT reagent kit with gDNA Eraser (Perfect Real Time, Takara, Japan). The quantity of cDNA for transcripts of *myf5*, *myod*, *myog*, *mrf4*, *mstn*, *igf-I*, *igf-II*, *igf1r*, *igf2r*, *murf-1*, *mafbx*, *capn1*, *capn2*, *ctsd*, *ctsl*, *opa1*, *mfn1*, *mfn2*, *tnf-α*, *il-6*, *gysm* and *pygm* were analyzed on the ABI7500 RT-PCR system (Applied Biosystems, USA) using TB Green Fast qPCR Mix (Takara, Japan). Relative quantifies of target genes were calculated by the ΔΔCt method using *β-actin* gene expression as reference. All the primers used in present study were listed in Table 5.

**Table 5:** List of PCR primer pairs used for the real-time PCR analysis.

Genes	Forward (5′–3′)	Reverse (5′–3′)	Accession no.
*myf5*	GCAACGCCATCCACTACATCG	TGCATTCAACTGGTGCCACACT	DQ872515
*myod*	GCAACGCCATCAGCTACATCG	CGTTTGGAGTCTGGGAGAAATAAG	DQ184914
*myog*	GTCTGGGGGTGTTGGAGTTGG	GACGCCTCTTCTCCCTCATCG	EF144128
*mrf4*	AGAGCAGCGGGGAGGAACAC	GACCTTGCAGGCCCACATGA	MK453386
*mstn*	TTTGAGGACTTTGGCTGGGACT	GCGACATCTTGGTGGGGGTA	DQ412048
*igf-I*	CTGTGCACCTGCCAAGACTA	CTTTGTGCCCTGCGGTACTA	MK453382
*igf-II*	ATCAAAGCACAGGAGCAGGCAATC	TGTCCGTGGCGAGCAAGACG	MK453383
*igf1r*	GAAGGGCGTGGTCAAAGATG	AGGTCGGAGGGAGCGTAAGT	MK453384
*igf2r*	TCCGCTGGTACACGTCCTAC	GGTGAGCCCTGATCCGATAT	MK453385
*murf-1*	TTGTGCCGTAGTTGTGCTAGTGAC	CATGGCGATCAAGCACGACCTC	MK292717
*mafbx*	GCTGGGTGAAAACCGAGGAG	CTTCTTGGCAGCCATGTCGT	MK453387
*capn1*	CATCGTAGACGGAGCCACTC	GACCGTGAGGAACCACTCTG	MK292720
*capn2*	AAAGTGAACGGCTGCTACGA	TCGTAGTTCTCAGCGATGCC	MK453381
*ctsd*	ACGTGCACAATCGGAGACTT	GATGTTGTCAAAGACCGGCG	FJ172450
*ctsl*	CTCCTGCTGGTCCTTCAGTTCAAC	ACGATGTAGCGGAAGGCATTGTC	FJ172449
*opa1*	CAGTGGCCGAGAGTTTGACC	TCACCGTACTGATGACGCCT	MK757585
*mfn1*	CGGTATTGGCCACACCACTA	AGAGCCCTCTGTCTTGAGGT	MK757584
*mfn2*	TGGTGACAGGTCTTGCATCC	CAACCCACTGCCTTCCAGAT	MK757586
*tnf-α*	GTCCTGGCGTTTTCTTGGTA	CTTGGCTCTGCTGCTGATTT	AB040448
*il-6*	CTCCAGTCGAATACGAGCCC	ACTCTTTCTGGTGGTGAGCG	DQ884914
*gysm*	GAGGAGCACATAGCAGACCC	TTACACGACTCATCGACCGC	MN201568
*pygm*	AACAATGACCGAGTGGTGGG	TTCTCAGCCAGAGTGACACG	MN201569
*β-actin*	GGAAATCGTGCGTGACATTAAG	CCTCTGGACAACGGAACCTCT	HQ386788

To attain the partial sequences of *mrf4*, *igf-I*, *igf-II*, *igf1r*, *igf2r*, *murf-1*, *mafbx*, *capn1*, *capn2*, *opa1*, *mfn1* and *mfn2* in olive flounder, primers were designed by Primer 5 (Primer, Canada) based on the conserved regions of cDNA sequences in other fish species available at the GenBank website (https://www.ncbi.nlm.nih.gov/genbank/). The detailed procedure was according to the previously description^124^.

### Western blot analysis

The frozen muscle samples (100 mg) were lysed in radioimmunoprecipitation lysis buffer (cat. No. R0020, Beijing Solarbio Science and Technology Co., Ltd., Beijing, China) supplemented with protease and phosphatase inhibiter cocktail (Roche, USA). Homogenates were centrifuged at 12,000×*g* for 10 min at 4 ℃, and the protein concentration in the supernatant was determined using a Bicinchoninic Acid Protein assay kit (cat. No. P0009, Beyotime Institute of Biotechnology, Shanghai, China). Equal amounts of protein were separated by sodium dodecyl sulfate–polyacrylamide gels (SDS-PAGE) and transferred to 0.45 μm PVDF membrane (Millipore, USA). Incubation with the primary antibody was performed overnight at 4 ℃. The primary antibodies used were phosphor-AMPK (Thr172) (dilution 1:1000, Beyotime Institute of Biotechnology, cat. No. AA393), AMPK (dilution 1:1000, EnoGene Biotech, Cat. No. E1A003B), phospho- S6 (Ser235/236) (dilution 1:4000, CellSignaling Technology Inc., Cat. No. 4858), S6 (dilution 1:2000, CellSignaling Technology Inc., Cat. No. 2217), phospho-mTOR (Ser2448) (dilution 1:1000, CellSignaling Technology Inc., Cat. No. 2971), mTOR (dilution 1:2000, CellSignaling Technology Inc., Cat. No. 2972), PGC-1α (dilution 1:1000, Abcam, Cat. No. ab54481), GLUT4 (dilution 1:1000, WanleiBio, Cat. No. WL02425) and β-actin (dilution 1:5000, Bioss Antibodies, Cat. No. bs-0061R). After the incubation, the membrane was washed with TBST and incubated with secondary antibody (HRP-labeled goat anti-Rabbit lgG) (Beyotime Institute of Biotechnology, Shanghai, China) at 1:5000 dilution for 1 h at room temperature. After that, the membrane was developed with Beyo ECL Plus reagents (Beyotime Institute of Biotechnology, Shanghai, China) and exposed to the X-ray file. The band densities were quantified using NIH Image 1.6α3 software and normalized to that of αβ-actin.

### Statistical analysis

All statistical evaluations were analyzed by one-way analysis of variance (ANOVA) followed by Tukey’s multiple range tests using the software SPSS 22.0. All data were expressed as means ± SE. Differences were considered significant when *P* < 0.05.

## Supplementary information

Supplementary Information.

## Data Availability

The data used to support the findings of the present study are available from the corresponding author on reasonable request.
