# Supplementary material for: Dietary carbohydrates influence muscle texture of olive flounder Paralichthys olivaceus through impacting mitochondria function and metabolism of glycogen and protein
Source: Sci Rep. 2020 Dec 11;10:21811. doi: 10.1038/s41598-020-76255-3 (PMC7732841; doi:10.1038/s41598-020-76255-3)
Supplement: Supplementary file 1 — Supplementary Information. [file 41598_2020_76255_MOESM1_ESM.docx]

**Dietary carbohydrates influence muscle texture of olive flounder *Paralichthys olivaceus* through impacting mitochondria function and metabolism of glycogen and protein**

Supplementary information

Jiahuan Liu^1^, Kangyu Deng^1^, Mingzhu Pan^1^, Guangxia Liu^1^, Jing Wu^1^, Mengxi Yang^1^, Dong Huang^1^, Wenbing Zhang^1, 2 *^, Kangsen Mai^1, 2^

*^1^ The Key Laboratory of Aquaculture Nutrition and Feeds (Ministry of Agriculture and Rural Affairs); the Key Laboratory of Mariculture (Ministry of Education), Ocean University of China, Qingdao 266003, China.*

*^2^ Laboratory for Marine Fisheries Science and Food Production Processes, Qingdao National Laboratory for Marine Science and Technology, Wen Hai Road, Qingdao 266237, China.*

* Corresponding author: Wenbing Zhang

*E-mail address*: wzhang@ouc.edu.cn (W. Zhang).

**Table S1.** Survival and growth performance of olive flounder fed with experimental diets. All data were expressed as mean ± SE. Mean values within the same row with different superscripts are significantly different (*P* < 0.05; Tukey's test).

|  | Dietary carbohydrate level, % dry matter | | | |  |  |
| --- | --- | --- | --- | --- | --- | --- |
|  | 0 | 8 | 12 | 16 | 20 | 24 |
| Initial weight (g) | 7.06±0.01 | 7.19±0.06 | 7.15±0.03 | 7.19±0.08 | 7.15±0.04 | 7.10±0.10 |
| Final weight (g) | 91.39±1.34^b^ | 78.92±1.28^a^ | 90.33±1.32^b^ | 92.84±1.44^b^ | 89.98±1.38^b^ | 83.96±1.18^ab^ |
| SR (%) | 94.22±1.18 | 94.89±2.13 | 93.33±0.67 | 94.89±0.59 | 95.11±1.74 | 95.56±0.98 |
| SGR (% /d) | 3.57±0.07^b^ | 3.42±0.10^a^ | 3.62±0.06^bc^ | 3.65±0.06^c^ | 3.61±0.06^bc^ | 3.52±0.10^b^ |
| FER | 1.42±0.02 | 1.39±0.01 | 1.41±0.02 | 1.42±0.02 | 1.39±0.03 | 1.38±0.02 |

Survival rate (SR, %) = 100 × (final fish number/initial fish number)

Specific growth rate (SGR, %) = [(ln final body weight − ln initial body weight) × 100/days

Feed efficiency ratio (FER) = wet weight gain (g)/dry feed fed (g)


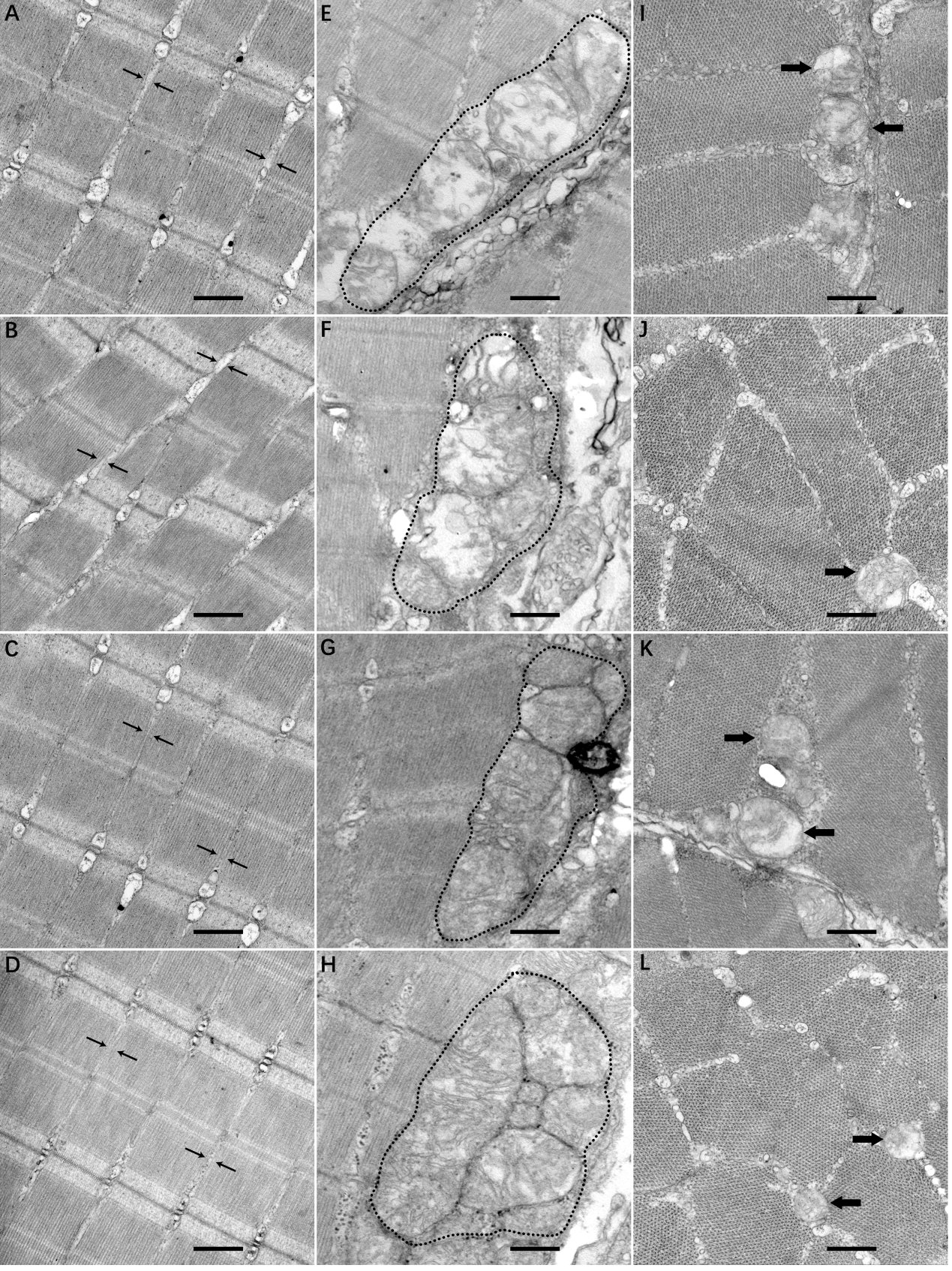


**Figure S1.** **Ultra-thin section of skeletal muscle from the C8, C12, C16 and C20 groups, bar=500nm.** Little glycogen granules between the myofibrils in the C8 (A), C12 (B), C16 (C) and C20 (D) groups. Mitochondria rather than glycogen granules are seen near the endomysium in the C8 (E), C12 (F), C16 (G) and C20 (H) groups. Normal mitochondrion in fish muscle in t the C8 (I), C12 (J), C16 (K) and C20 (L) groups.


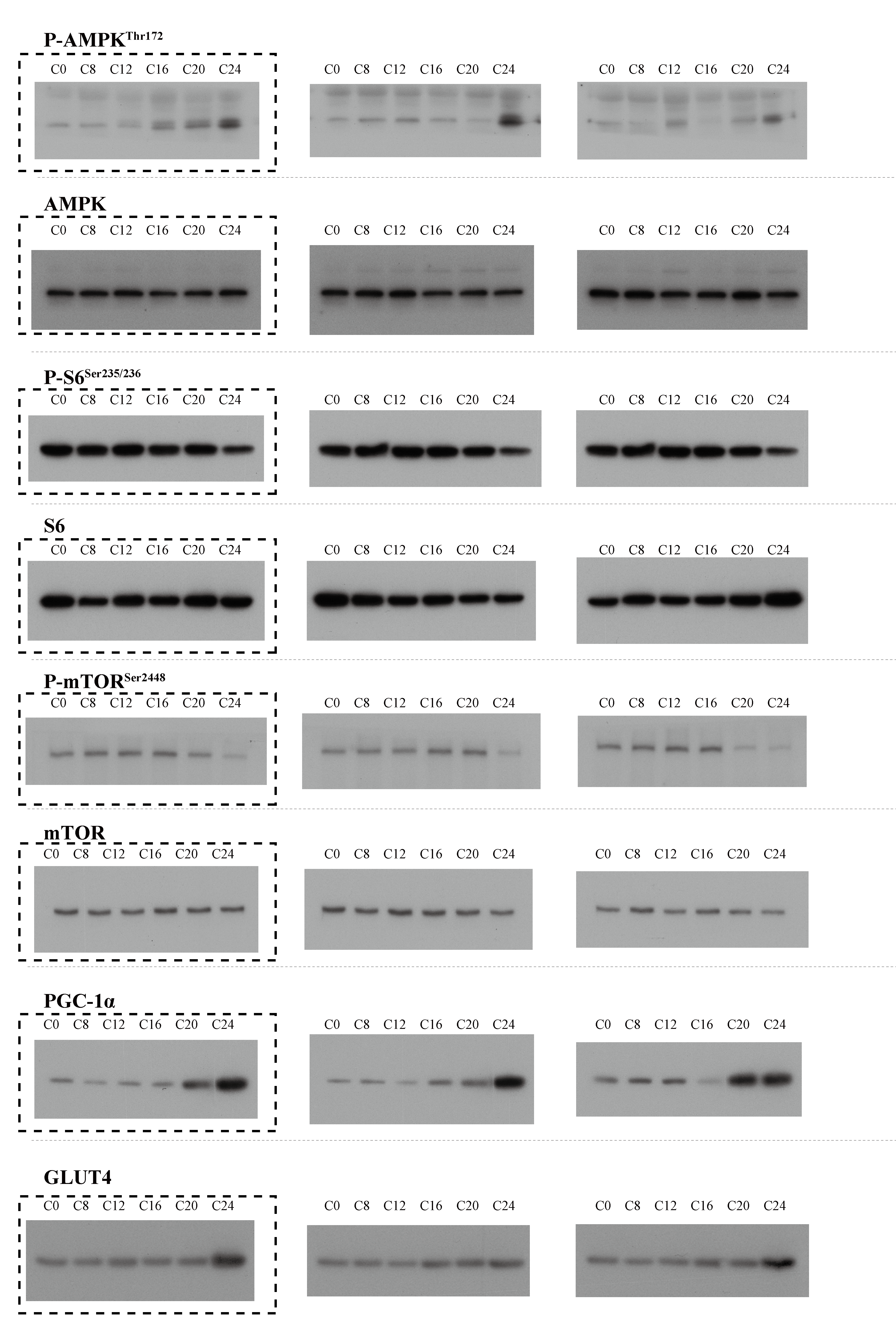


**Figure S2. The original, unprocessed versions of western blot images.** The different original images correspond to different biological replicates. The framed original blots have been used to prepare the cropped blots shown in Figure 4.
